# Meta-Analytic Review of High Anxiety Comorbidity among Patients with Vitiligo

**DOI:** 10.1155/2021/6663646

**Published:** 2021-05-17

**Authors:** Jiani Liu, Rui Tang, Yangfan Xiao, Mei Luo, Yaqian Shi, Qiancheng Deng, Huiming Zhang, Zhoutong Zeng, Zixin Pi, Rong Xiao

**Affiliations:** ^1^Department of Dermatology, The Second Xiangya Hospital, Central South University, Changsha, Hunan 410011, China; ^2^Department of Rheumatology and Immunology, The Second Xiangya Hospital, Central South University, Changsha Hunan 410011, China; ^3^Department of Anesthesiology, The Second Xiangya Hospital, Central South University, Changsha, Hunan 410011, China; ^4^Department of Neurosurgery, Xiangya Hospital, Central South University, Changsha, Hunan 410008, China

## Abstract

**Background:**

Vitiligo is a disfiguring skin disease with profound psychosocial impacts, such as anxiety, but the reported effect sizes of associations vary. We aimed to conduct a meta-analysis to quantify the strength of association between anxiety and vitiligo and to estimate the prevalence of anxiety among individuals with vitiligo.

**Methods:**

A systematic literature search was performed in five online databases (MEDLINE, Embase, Web of Science, Cochrane Library, and PsycINFO) from inception until March 20, 2020. All of the eligible studies were comprehensively reviewed, and all of the available data were analyzed according to our predefined criteria.

**Results:**

Twenty-one studies involving 3259 patients in 11 countries were included in this meta-analysis. Compared with the healthy control group, patients with vitiligo often had concomitant anxiety (OR = 6.14 [95% CI: 3.35–11.24], *I*^2^ = 30.1%). The pooled prevalence of anxiety in female patients was significantly higher than that in males (OR = 2.24 [95% CI: 1.31–3.84], *I*^2^ = 0.0%). Subgroup analysis showed that the pooled prevalence of clinical anxiety disorder and anxiety symptoms was 12% (95% CI: 7%–16%, *I*^2^ = 76.3%) and 34% (95% CI: 21%–46%, *I*^2^ = 94.7%), respectively. No publication bias has been detected by Begg's funnel plot and Egger's test.

**Conclusion:**

Patients with vitiligo have high anxiety comorbidity, with female predominance. Dermatologists and psychiatrists should be vigilant to the presence of anxiety, apply appropriate interventions to reduce the psychological impacts in a timely manner, and thus promote recovery in vitiligo patients. However, due to some objective limitations (poor information about the OR and diversity in assessment tools among included studies), findings should be interpreted with caution.

## 1. Introduction

Vitiligo is an autoimmune skin disease characterized by depigmented macules, which result from progressive melanocyte destruction in the epidermal area [1]. It is estimated that 0.5%–2% of the global population suffers from vitiligo [2, 3]. Its prevalence varies between different regions. As skin lesions of vitiligo often occur in the exposed areas of the skin, patients with vitiligo often suffer from devastating disfigurement, social discrimination, and a subsequent psychological burden [4]. A large-sample study reported that 27.49% of inpatients with vitiligo in the US are concomitantly diagnosed with mental health disturbances [5]. A poor mental health state can impair the quality of life and impede the treatment of vitiligo. Moreover, it has been inferred that vitiligo can be triggered by psychological factors, such as excessive stress [6]. Therefore, it is widely accepted that vitiligo should be redefined as a psychodermatologic disease [7].

However, the evaluation of the mental health state in individuals with vitiligo has not been well demonstrated. On the one hand, most dermatologists focus on the treatment of skin lesions and ignore the potential mental symptoms. On the other hand, the majority of vitiligo patients reject the proposal for referral to a psychiatrist due to the stigma associated with psychosis [8]. Recently, dermatologists have started to assess the prevalence of depression and subsequent suicide. Nonetheless, the screening, diagnosis, and treatment of anxiety in patients with vitiligo lag far behind in practice. Based on current research, the prevalence of anxiety in patients with vitiligo varies from 4% to 62% [9, 10], depending on multiple factors, such as the study design, the used outcome assessment tool, the definition of anxiety, the study population, and the sample size. Although a previous meta-analysis reported the prevalence of anxiety in vitiligo patients by clinical diagnosis criteria and anxiety-specific questionnaires [11], it remains controversial whether vitiligo patients have a higher risk of anxiety.

Recently, numerous studies in this area have been published. Therefore, an up-to-date meta-analysis is essential to analyze the relationship between anxiety and vitiligo.

## 2. Materials and Methods

### 2.1. Literature Search Strategy

This meta-analysis was based on the Preferred Reporting Items for Systematic Review and Meta-Analysis (PRISMA) guidelines [12]. We followed the methods of Zou et al. [13]. We conducted a comprehensive online search in MEDLINE (PubMed), Web of Science, Embase, the Cochrane Library, and PsycINFO (Ovid). The search strategy included the following terms: (1) “vitiligo” or “hypopigmentation” or “depigmentation” or “leukoderma” and (2) “anxiety disorders” or “anxiety” or “social anxiety” or “hypervigilance” or “nervousness.” Articles were published before March 20, 2020. The abstract and full text were screened according to the inclusion and exclusion criteria. The references of all of the included studies were conditionally screened.

### 2.2. Eligibility Criteria and Article Selection

Original articles matched the following inclusion criteria: (1) cross-sectional, case-control, or cohort study design; (2) a sample of patients clinically diagnosed with vitiligo; (3) anxiety was clinically diagnosed or evaluated by specific questionnaires; (4) sufficient raw data were available for analysis; and (5) manuscript in English or Chinese. Exclusion criteria were as follows: (1) reviews, conference abstracts, letters, or case reports and (2) duplicated or overlapping data. The process of article selection was conducted by two researchers (Jiani Liu and Rui Tang) independently. Any discrepancies were resolved by mutual discussion.

### 2.3. Data Extraction and Quality Assessment

Two independent researchers (Jiani Liu and Rui Tang) extracted data from the included studies. The following baseline information were extracted: first author's name, year of publication, study design, country, race of participants, number of participants, gender information, age, anxiety scales, and quality assessment score. To accurately evaluate the quality of each eligible study, all of studies were evaluated by the Newcastle–Ottawa Scale (NOS) [14, 15]. The quality assessment of each study was based on the participant selection process, comparability, outcome ascertainment, and data processing. A score greater than 7 indicated high quality, a score of 4–7 represented moderate quality, and a score of less than 4 was classified as poor quality.

### 2.4. Statistical Analysis

All of the data processing and analysis were performed using Stata version 15.0 (StataCorp, College Station, TX, USA). The association between anxiety and vitiligo was estimated by the odds ratio (OR) for dichotomous data and standard mean differences (SMDs) for continuous data, with their corresponding 95% confidence intervals (CIs). The chi-squared test and *I*^2^ statistics were applied to identify heterogeneity among studies. A fixed-effects model was applied to estimate the pooled effect size in the case of no significant heterogeneity (*I*^2^ < 50% or *P* > 0.1); otherwise, the DerSimonian–Laird random-effects model was employed [16]. To explore potential sources of heterogeneity between studies, sensitivity analysis and subgroup analysis were conducted. Egger's test and Begg's test were used to detect publication bias [17].

## 3. Results

### 3.1. Literature Search and Study Selection

**Figure 1:**
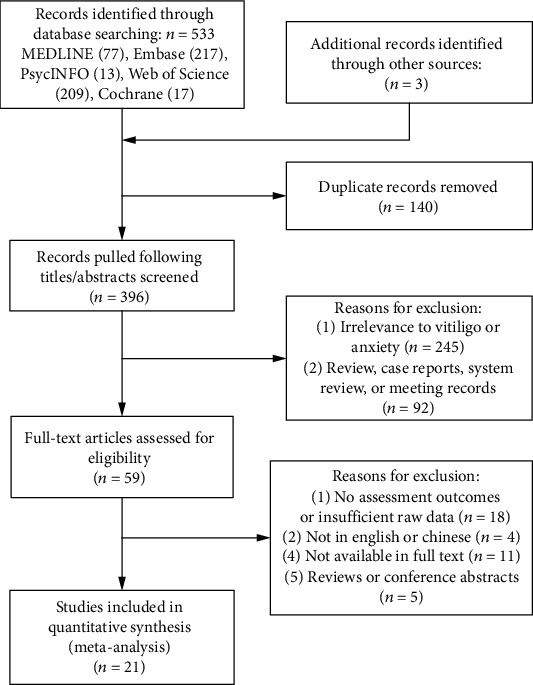
Flow diagram of literature selection strategy.

A total of 533 publications were identified from the primary online search (MEDLINE 77, Embase 217, the PsycINFO 13, Web of Science 209, and the Cochrane Library 17). Three additional articles were included through reference tracking. Next, 140 duplicates were removed. 337 articles were excluded due to irrelevant topics after screening of the titles and abstracts. After a meticulous full-text review of the remaining articles, 38 articles were excluded. Eventually, 21 studies involving 3259 cases were included for meta-analysis. Details of the online search strategy are presented in Figure 1.

### 3.2. Characteristics of Included Studies

The main information of the 21 included studies is presented in Table 1. Nineteen articles were in English and two were in Chinese. Thirteen cross-sectional studies, seven case-control studies, and one cohort study were included. All of the studies were published between 2001 and 2020 in 11 different countries. All of the vitiligo patients were recruited by dermatology clinics (*n* = 20) or retrieved from the online database (*n* = 1). The sample sizes of participants ranged from 24 to 1432 people, and gender information was also presented. The mean age in all of the studies except for that by Ucuz et al. [18] ranged from 24.6 to 47.1 years. Ten screening scales were applied to evaluate the prevalence of anxiety. Nineteen studies reported the prevalence of anxiety in vitiligo patients, and six studies reported the prevalence in both vitiligo patients and healthy controls. Thirteen studies had a quality score of 7 or higher, suggesting an overall good quality of the included studies.

### 3.3. The Strength of Association between Anxiety and Vitiligo

**Figure 2:**
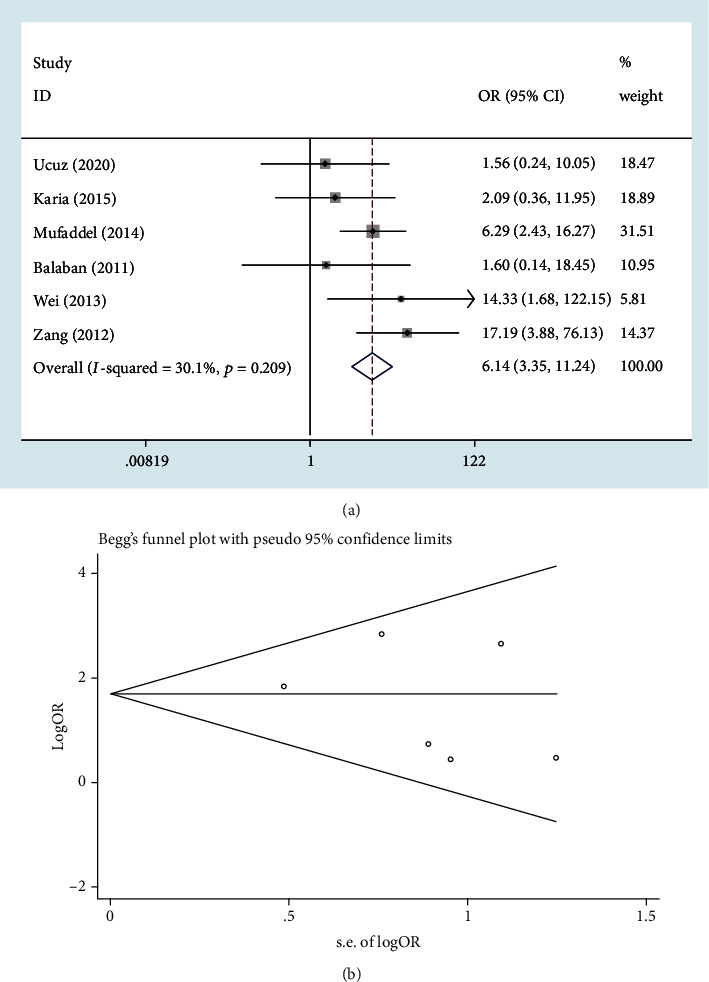
The strength of association between anxiety and vitiligo: (a) Forest blot; (b) Begg's funnel plot.

Six case-control studies reported data about the prevalence of anxiety in both vitiligo patients and healthy controls [9, 10, 18–21]. The pooled OR for the prevalence of anxiety between the two groups was 6.14 (95% CI: 3.35–11.24; *P* < 0.001), which demonstrated the association between anxiety and vitiligo. No significant heterogeneity has been revealed (*I*^2^ < 30.1%; *P* = 0.209) (Figure 2(a)). Begg's test (Pr > ∣*z* | = 0.707), Egger's test (*P* > ∣*t* | = 0.514), and the funnel plot illustrated that no publication bias was detected (Figure 2(b)).

### 3.4. Female Predominance of Anxiety in Patients with Vitiligo

**Figure 3:**
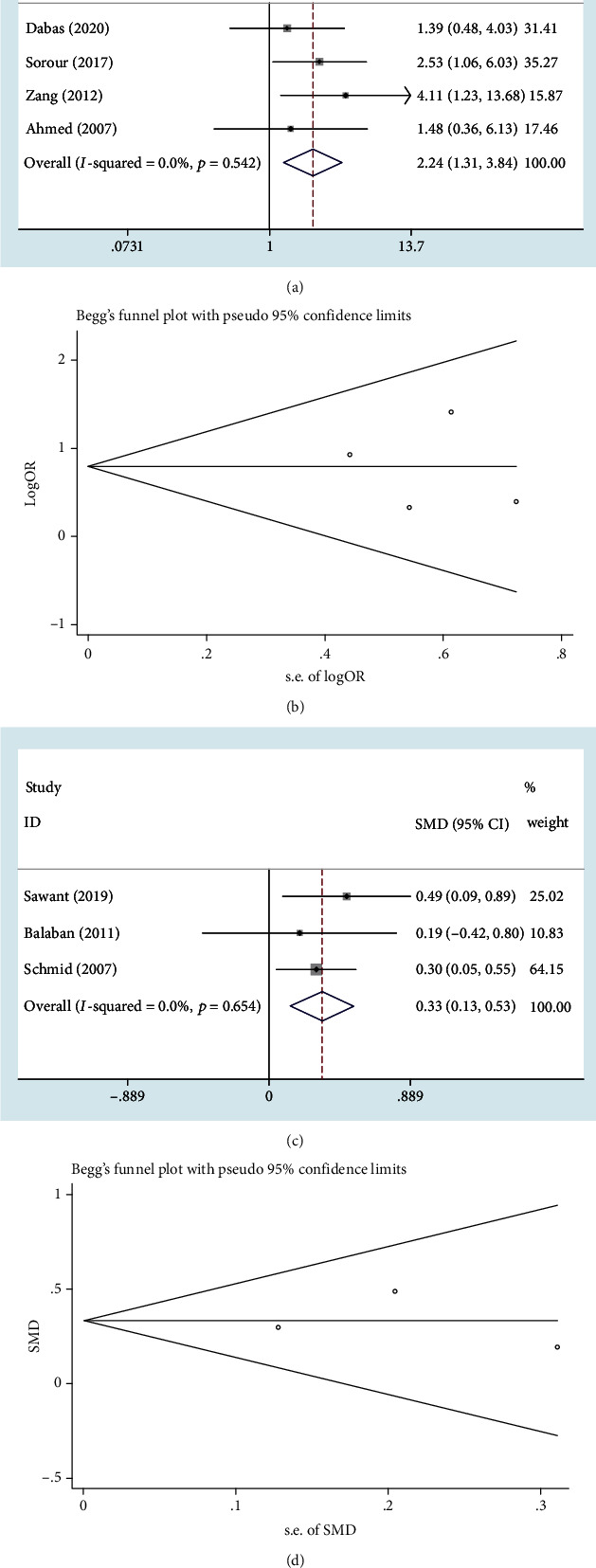
Female predominance of anxiety in patients with vitiligo: (a, c) Forest plot; (b, d) Begg's funnel plot.

The prevalence of anxiety in male and female patients with vitiligo was separately provided in four studies [8, 19, 22, 23]. The pooled OR of anxiety for female patients was 2.24 (95% CI: 1.31–3.84; *P* = 0.003) with no heterogeneity (*I*^2^ = 0.0%; *P* = 0.542) (Figure 3(a)). Begg's test (Pr > ∣*z* | = 1.000), Egger's test (*P* > ∣*t* | = 0.826), and the funnel plot detected no publication bias (Figure 3(b)). Moreover, three studies reported the mean value and standard deviation of social anxiety for males and females separately [9, 24, 25]. Pooled results (SMD = 0.33, 95% CI: 0.13–0.53; *P* = 0.001) suggested that female patients were more susceptible to social anxiety (Figure 3(c)). No heterogeneity was observed in the analysis (*I*^2^ = 0.0%; *P* = 0.654). No publication bias was detected by Begg's test (Pr > ∣*z* | = 1.000), Egger's test (*P* > ∣*t* | = 0.983), and the funnel plot (Figure 3(d)).

### 3.5. The Pooled Prevalence of Anxiety in Patients with Vitiligo

**Figure 4:**
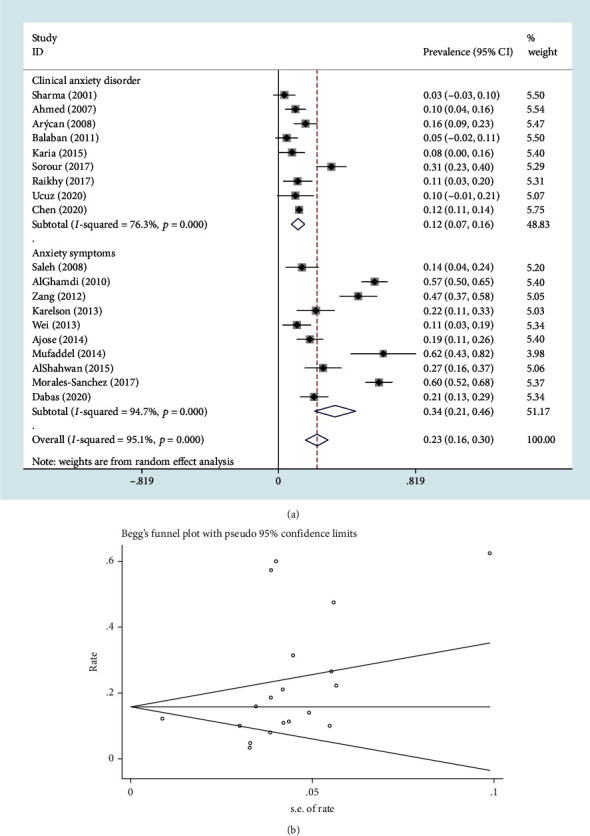
The pooled prevalence of anxiety in patients with vitiligo: (a) Forest plot; (b) Begg's funnel plot.

Nineteen studies [8–10, 18–23, 26–35] reported data about the prevalence of anxiety in patients with vitiligo via diversified outcome measurement tools. The pooled prevalence of anxiety was 23% (95% CI: 16%–30%) (Figure 4(a)). Egger's test (*P* > ∣*t* | = 0.060) and the funnel plot did not indicate any publication bias (Figure 4(b)). As considerable heterogeneity (*I*^2^ = 95.1%; *P* < 0.001) was observed for the pooled prevalence of anxiety, subgroup analysis was performed based on outcome measurement tools. The pooled prevalence of clinical anxiety disorders based on clinical diagnostic criteria among 9 studies [9, 18, 21–23, 26, 28, 33, 34] was 12% (95% CI: 7%–16%) with heterogeneity (*I*^2^ = 76.3%; *P* < 0.001), and that of anxiety symptoms based on valid screening scales among 10 studies [8, 10, 19, 20, 27, 29–32, 35] was 34% (95% CI: 21%–46%) with heterogeneity (*I*^2^ = 94.7%; *P* < 0.001).

### 3.6. Sensitivity Analysis

**Figure 5:**
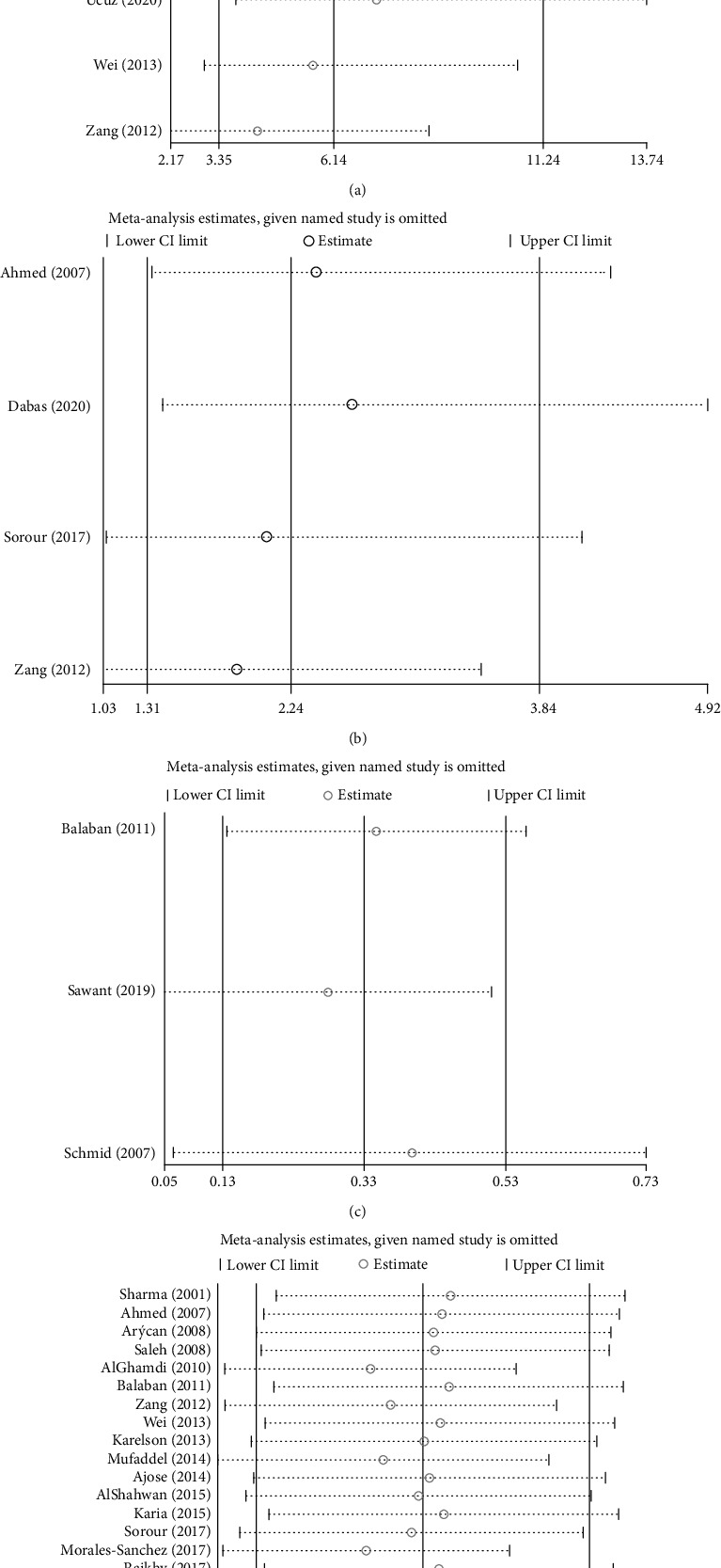
Sensitivity analysis: (a) for the strength of association between anxiety and vitiligo; (b) for the female predominance (OR) of anxiety in patients with vitiligo; (c) for the female predominance (SMD) of anxiety in patients with vitiligo; (d) for the pooled prevalence of anxiety in patients with vitiligo.

Sensitivity analysis was conducted to confirm the stability of all of the analyses with respect to the comorbidity of vitiligo and anxiety. The pooled results of all of the analyses were not significantly changed after the removal of any specific studies (Figure 5).

## 4. Discussion

Compared with the relevant study by Osinubi et al. [11], we firstly quantified the strength of association between anxiety and vitiligo; moreover, we clarified the difference in prevalence between genders. Our pooled results indicate that patients with vitiligo carry a substantial burden due to anxiety. The risk of anxiety in individuals with vitiligo was 6.14 times as high as that in healthy controls. Of vitiligo patients, 23% suffer from this psychological disorder, and the prevalence of anxiety is significantly higher in females than in males. However, further studies with standard anxiety-specific scales and larger sample sizes are essential to support our findings.

Plenty of clinical studies have reported the viewpoint that patients with vitiligo are susceptible to anxiety disorders. We first conducted a quantitative assessment of the susceptibility of anxiety in vitiligo patients. Several hypotheses may explain the increased prevalence of anxiety disorders among individuals with vitiligo. In some regions, skin depigmentation may be regarded as a sign of low social status, and patients with skin depigmentation suffer from more discrimination in daily life [36]. Patients with vitiligo experience more anxiety because they fear being stigmatized. Many patients consider skin depigmentation as an impenetrable barrier for finding a suitable job or getting married [37]. Such patients are at high risk of developing anxiety disorders and tend to present social avoidance.

Subgroup analysis was conducted to evaluate the influence of different cultures on the prevalence of anxiety among participants with vitiligo. Although the results did not achieve statistical significance, an interesting appearance was that the pooled prevalence of anxiety increased from 15% for studies conducted in Asians to 23% for that in the Middle East. This diversity might be related to different levels of vitiligo acceptance between different cultures [35]. However, due to the lack of sufficient sample size, the pooled prevalence from Africa, Europe, or North America participants was unmeasurable, separately (Figure S1).

Most viewpoints assumed that vitiligo patients with dark skin (Fitzpatrick skin phototypes IV-VI) are more susceptible to anxiety, depression, and other psychological diseases because of greater notable depigmented patches and the stigma [38, 39]. However, through a vitiligo-specific burden questionnaire based on skin phototype, Ezzedine et al. found that the psychological burden of vitiligo was similar in all patients with dark or fair skin. Specifically, dark-skinned patients cared more about the affected appearance, whereas fair-skinned individuals felt more anxious about skin cancer occurrence [40, 41].

This association between vitiligo and anxiety can partly be explained by molecular biological mechanisms. Both the skin and the brain originate from the ectoderm during embryogenesis and are regulated by many of the same hormones and neurotransmitters [42]. O'Sullivan et al. [42] have proposed the neuroimmune-cutaneous-endocrine model to explain the relationship between psychological factors and inflammatory skin diseases. The hypothalamic-pituitary axis responds to psychological stress with sympathetic nervous system activation and upregulation of stress hormones and neuromodulators like corticotropin-releasing hormone and substance P, leading to skin mast cell activation and Th1/Th2 immune response dysregulation, eventually inducing inflammatory skin disorders [43].

As far as we know, this study is the first meta-analysis to clarify the female predominance of anxiety in vitiligo patients. According to our data, females have a higher risk of anxiety and develop more severe social anxiety, which may be related to greater cosmetic awareness and lower self-confidence [25]. Generally, females present a more negative self-evaluation and have a problem adapting to skin diseases [24]. From a biological point of view, the hypothalamic-pituitary-adrenal (HPA) axis can regulate various anxiety-related hormones, such as oxytocin, prolactin, and GABA. Among females, the HPA axis tends to be more dysregulated when females are faced with excessive stress [44]. In addition, the marked fluctuations of gonadal hormones during premenstrual and postpartum periods are likely to contribute to the onset of anxiety-related symptoms in women [45].

We have observed notable heterogeneity between studies with respect to the pooled prevalence of anxiety, which may be due to the fact that different anxiety-specific scales and clinical diagnostic criteria were utilized in different studies. Six clinical diagnostic criteria (such as DSM-V, ICD-10, and K-SADS-PL) and 10 different validated screening scales were separately applied in the included studies, of which two scales (ACS-SAA and LSAS) were aimed at evaluating social anxiety. Therefore, we conducted a subgroup analysis to identify whether the difference in outcome assessment tools would be a potential heterogeneity source. Our results indicate that the prevalence identified by clinical criteria was significantly lower than that identified by screening scales. This can be explained by the fact that the purpose of screening scales is to identify as many anxiety symptoms or subclinical anxiety emotions as possible and to rate these manifestations; however, the purpose of clinical criteria is to accurately diagnose clinical anxiety disorders and to conduct medical interventions in a timely manner. In the subgroup analysis, the heterogeneity between studies was slightly lower. Differences in measurement standards among various screening scales were among the confounding factors that cannot be avoided. An interesting problem is that thresholds applied to screening scales to define anxiety symptoms also differed between studies. Even if the same scale was used, like HADS-A, the measurement thresholds to define outcomes differed between studies [10, 30, 31]. A universal screening scale for anxiety that is suitable for vitiligo patients specifically is unfortunately still lacking.

Additionally, depression, phobia, adjustment disorder, and somatoform disorder also occur in patients with vitiligo [8, 22, 46, 47]. Anxiety and depression are the most frequent psychological defects among vitiligo patients [11]. Compared to depression, anxiety disorder seems to be more prominent in young patients with vitiligo [18, 48]. Given that the severity and activity of diseases are positively correlated with the prevalence of anxiety, all dermatologists should put a premium on the impact of anxiety and other psychological burden on patients with vitiligo [8].

The limitations of our article are worth discussing. For example, the case-control studies comparing the prevalence of anxiety in vitiligo patients and healthy controls are limited. Only a small number of studies reported the prevalence of anxiety in female and male patients separately. Although no heterogeneity has been observed in these analyses, more related studies with more candidates are required to support our conclusion. Moreover, the high heterogeneity in the analysis of the prevalence of anxiety in vitiligo patients cannot be ignored. Besides the measurement tools, other factors might be taken into consideration for the heterogeneity, such as the study design and age of participants. Therefore, more results based on those confounding factors would be helpful to our further research.

## 5. Conclusions

In conclusion, comorbidity of anxiety and vitiligo is common in the clinic. Patients with vitiligo have an enormous burden due to anxiety, with female predominance. Dermatologists and psychiatrists should be vigilant to the presence of anxiety, apply appropriate interventions to reduce the psychological impacts in a timely manner, and thus promote recovery in vitiligo patients. A better designed case-control study and larger sample sizes are warranted for future studies. Moreover, in the field of psychodermatology, a uniform scale to measure anxiety in vitiligo patients is urgently needed.

## Figures and Tables

**Table 1:** Characteristics of included studies.

Study	Study design	Country	Participants	Gender (male %)	Age (mean)	Anxiety measurement tools (cut-off)	Anxiety in patients with vitiligo (%)	Quality assessment by NOS (score)
Sharma et al. (2001)	Cross-sectional	India	30 vitiligo and 30 psoriasis	17 (56.7%) vs. 18 (60.0%)	N/A	DSM-IV	3.3	6
Ahmed et al. (2007)	Cross-sectional	Pakistan	100 vitiligo	38 (38.0%)	24.6	PAS	10.0	5
Schmid-Ott et al. (2007)	Cross-sectional	Germany	363 vitiligo	79 (21.8%)	43.5	ACS-SAA	N/A	6
Saleh et al. (2008)	Cross-sectional	Egypt	50 vitiligo and 50 psoriasis	25 (50.0%) vs. 25 (50.0%)	28.5 vs. 38.2	TMAS	14.0	6
Arýcan et al. (2008)	Cross-sectional	Turkey	113 vitiligo	53 (46.9%)	M: 29.2, F: 33.4	Psychiatrists	15.9	7
AlGhamdi (2010)	Cross-sectional	Saudi Arabia	164 vitiligo	91 (55.5%)	27	IPQ	57.0	6
Ajose et al. (2014)	Cross-sectional	Nigeria	102 vitiligo and 87 albinos	51 (50.0%) vs. 53 (60.9%)	35.94 vs. 30.05	HADS-A (>10)	18.6	9
AlShahwan (2015)	Cross-sectional	Saudi Arabia	64 vitiligo and 811^a^	N/A	N/A	HADS-A (>10)	26.6	6
Morales-Sanchez et al. (2017)	Cross-sectional	Mexico	150 vitiligo	47 (31.3%)	N/A	BAI (>15)	60.0	8
Raikhy et al. (2017)	Cross-sectional	India	53 vitiligo and 947^a^	N/A	N/A	ICD-10	11.3	7
Sorour et al. (2017)	Cross-sectional	Egypt	108 vitiligo and 934^a^	48 (44.4%) vs. N/A	N/A	DSM-V	31.5	9
Sawant et al. (2019)	Cross-sectional	India	100 vitiligo	56 (56.0%)	M: 35.8, F: 36.9	ASC-SAA	N/A	8
Dabas et al. (2020)	Cross-sectional	India	95 vitiligo and 86 melasma	34 (35.8%) vs. N/A	N/A	GAD-7 (>8)	21.1	9
Chen et al. (2020)	Cohort	China	1432 vitiligo and 5728^b^	559 (39.0%) vs. 2239 (39.1%)	47.08 vs. 46.09	ICD-9-CM	12.2	8
Balaban et al. (2011)	Case control	Turkey	42 vitiligo and 33 HCs	19 (45.2%) vs. 14 (42%)	39.70 vs. 35.12	DSM-IV, LSAS	4.8	8
Zang and Ji (2012)	Case control	China	80 vitiligo and 40 HCs	33 (41.3%) vs. 16 (40.4%)	29.1 vs. 29	SAS (>50)	47.5	8
Karelson et al. (2013)	Case control	Estonia	54 vitiligo and 57 HCs	22 (40.7%) vs. 23 (40.4%)	36.6 vs. 39.7	ES-Q (>12)	22.0	7
Wei et al. (2013)	Case control	China	55 vitiligo and 118 HCs	29 (52.7%) vs. 60 (50.8%)	40.98 vs. 40.56	HAMA (>14)	10.9	5
Mufaddel and Abdelgani (2014)	Case control	Sudan	24 vitiligo and 105 HCs	N/A	N/A	HADS-A (>8)	62.5	7
Karia et al. (2015)	Case control	India	50 vitiligo and 50 HCs	22 (44.0%) vs. N/A	33.6 vs. N/A	DSM-IV	8.0	7
Ucuz et al. (2020)	Case control	Turkey	30 vitiligo and 30 HCs	18 (60%) vs. 18 (60%)	12.3 vs. 13.3	K-SADS-PL	10.0	6

Abbreviations: N/A: not applicable; HCs: healthy controls; M: males; F: females; NOS: Newcastle–Ottawa Scale; AHRQ-11: 11 Agency for Healthcare Research and Quality; DSM-IV: Diagnostic and Statistical Manual of Mental Disorders, 4th Edition; PAS: Psychiatric Assessment Schedule; ACS-SAA: Adjustment to Chronic Skin Diseases Questionnaire-Social Anxiety/Avoidance; TMAS: Taylor Manifest Anxiety Scale; IPQ: Illness Perception Questionnaire; LSAS: Liebowitz Social Anxiety Scale; SAS: Self-Rating Anxiety Scale; ES-Q: Emotional State Questionnaire; HAMA: Hamilton Anxiety Scale; HADS-A: Hospital Anxiety and Depression Scale-Anxiety; BAI: Beck's Anxiety Inventory; ICD-10: International Classification of Diseases, 10th Edition; DSM-V: Diagnostic and Statistical Manual of Mental Disorders, 5th Edition; ICD-9-CM: International Classification of Diseases, 9th Revision, Clinical Modification; GAD-7: General Anxiety Disorder-7; K-SADS-PL: Schedule for Affective Disorders and Schizophrenia for School Age Children-Present and Lifetime Version. ^a^Nonvitiligo patients with skin diagnosis; ^b^patients without vitiligo.

## Data Availability

The data supporting this meta-analysis are from previously reported studies and datasets, which have been cited. The processed data are available from the corresponding author upon request.

## Supplementary Materials

Supplementary Materials Figure S1: subgroup analysis of the influence of different regions on the pooled prevalence of anxiety among patients with vitiligo. The result is shown in the Forest plot.
