# Supplementary material for: Meta-Analytic Review of High Anxiety Comorbidity among Patients with Vitiligo
Source: Biomed Res Int. 2021 May 17;2021:6663646. doi: 10.1155/2021/6663646 (PMC8147524; doi:10.1155/2021/6663646)
Supplement: Supplementary Materials — Figure S1: subgroup analysis of the influence of different regions on the pooled prevalence of anxiety among patients with vitiligo. The result is shown in the Forest plot. [file 6663646.f1.docx]

## Supplementary Materials :

**Figure S1.** Subgroup analysis of the influence of different regions on the pooled prevalence of anxiety among patients with vitiligo. The result showed in the forest plot.
